# High-fat diet mouse model receiving L-glucose supplementations propagates liver injury

**DOI:** 10.3389/fnut.2024.1469952

**Published:** 2024-12-13

**Authors:** Johnny Amer, Athar Amleh, Ahmad Salhab, Yuval Kolodny, Shira Yochelis, Baker Saffouri, Yossi Paltiel, Rifaat Safadi

**Affiliations:** ^1^Liver Institute, Hadassah-Hebrew University Hospital, Jerusalem, Israel; ^2^Applied Physics Department, Center for Nanoscience and Nanotechnology, Hebrew University Givaat Ram, Jerusalem, Israel

**Keywords:** D-glucose, GLUT-2, HFD-fed mice, l-glucose, MASLD

## Abstract

**Background and aims:**

Limited data link manufactured sweeteners impact on metabolic dysfunction-associated steatotic liver disease (MASLD). We aimed to evaluate the effects of manufactured sugars (L-glucose) compared to natural sugars (D-glucose) on phenotype, molecular and metabolic changes in mice models fed with either regular diet (RD) or high fat diet (HFD).

**Methods:**

C57BL/6 mice fed 16-weeks with either RD; 70% carbohydrate or HFD; 60% fat, with or without additional glucose (Glu, at 18% w/v) to drinking tap water at weeks 8–16; of either natural (D-Glu) or manufactured (L-Glu) sugars. Liver inflammation (ALT and AST serum levels, liver H&E histologic stains and cell viability profile by p-AKT), liver fibrosis [quantitated *α* smooth-muscle-actin (αSMA) by western blot and RT-PCR, Masson Trichrome staining (MTC) of liver tissue], liver lipid [steatosis stain by H&E, Adipose Differentiation-Related Protein (ADRP) lipid accumulation, serum and lipid peroxidation Malondialdehyde (MDA) markers by ELISA], glucose hemostasis (serum Glucose and C-peptide with HOMA-IR score calculation) and liver aspects [hepatic glucose transporter 2 (GLUT2), insulin receptor (IR) expressions and GYS2/PYGL ratio] evaluated.

**Results:**

D- and L-Glu supplementations propagate hepatocytes ballooning and steatosis in HFD-fed mice and were associated with αSMA down-expressions by 1.5-fold compared to the untreated group while showed an acceleration in liver fibrosis in the RD-fed mice. Lipid profile (Steatosis, ADRP and MDA) significantly increased in HFD-fed mice, both Glu supplementations (mainly the L-Glu) increased serum MDA while decreased ADRP. HOMA-IR score and IR significantly increased in HFD-fed mice, with further elevation in HOMA-IR score following Glu supplementations (mainly L-Glu). The increase in HOMA-IR negatively correlated with IR and Glut2 expressions. D- and L-Glu supplementations showed significant decrease of Glycogenesis (low GYS2/PYGL ratio) and unchanged p-AKT pattern compared to their RD counterparts.

**Conclusion:**

Our data indicate an increase in rate of de-novo lipogenesis (DNL) in RD-fed mice (High carbohydrate diet) and liver fibrosis following additional sugar supplementations. In contrast, HFD-fed mice (with pre-existing high lipid profile) supplemented with sugar showed less liver fibrosis, because of reduced de-novo fatty acids synthesis and subsequently, the lipid oxidation pathways become dominated and induce the net results of lipid clearance.

## Introduction

Metabolic dysfunction–associated steatotic liver disease (MASLD) has become a prevalent health concern in the modern world, affecting up to 35–50% of the adult population and up to 20% of children (1). This condition, characterized by the accumulation of fat in the liver cells, occurs without significant alcohol consumption and is often linked to unhealthy lifestyle choices, including poor diet and lack of exercise (2).

Given the association between unhealthy lifestyle choices and the development of MASLD (3), it is crucial to examine how specific dietary components, such as different forms of glucose, might influence the disease’s progression (4). D-glucose (D-Glu), the naturally occurring form, is a fundamental carbohydrate in human metabolism, participating in essential processes such as glycolysis and the citric acid cycle (5). High levels of D-Glu, particularly in the form of high-fructose corn syrup and other sweeteners, can lead to increased expression of glucose transporters GLUT2, further promoting glucose and contributing to MASLD progression (6). Conversely, L-glucose (L-Glu); a Non-Nutritive Sugars (NNS), is a synthetic stereoisomer of glucose, and not metabolized by the body in the same manner as D-Glu (7). Due to its unique structure, L-Glu is poorly absorbed in the intestines and does not contribute significantly to caloric intake or blood glucose levels (7, 8). This raises intriguing questions about NNS potential impact on liver metabolism and MASLD as a dietary supplement.

A high-carbohydrate diet can prime the hepatic de-novo lipogenesis (DNL) pathway (9). DNL has been suggested to be abnormally increased in and contribute to the pathogenesis of MASLD (10), a highly prevalent metabolic disease that is linked to the development of type 2 diabetes mellitus (T2DM) (11).

One area of interest in MASLD research is dietary sugars’ role in the disease’s development and progression. While much attention has been given to the impact of natural sugars such as D-Glu, the effects of synthetic sugars like L-Glu still need to be explored. Our study aims to investigate the metabolic and phenotypic consequences of L-Glu supplementation compared to D-Glu in a high-fat diet (HFD) mouse model.

## Methods

### High fat diet animal model

The *in vivo* experiment was performed according to the regulations and guidelines of the National Institutes of Health (NIH) and the Hebrew University of Jerusalem under a protocol approved by the animal facility at the Hebrew University of Jerusalem with ethic number MD-18-154943. Six-week-old C57BL/6 J male mice (*n* = 60) were purchased from Harlan Laboratory, Jerusalem. Mice were placed on either a regular diet (RD) of isocaloric low-fat control diet (10% kcal energy from fat, 20% protein, and 70% carbohydrates) (Cat. # D12450B) or high-fat diet (HFD) (60% kcal energy from fat, 20% protein, and 20% carbohydrates) (Cat. # D12492) for 16 weeks. From week 8 to week 16, mice were watered with either tap water or 18% (w/v) D-Glu or 18% (w/v) L-glucose in RD and HFD mice groups. Mice’s initial maternal body weight and weekly weights and total food intake were recorded each week during the experiment. By week 16, mice were sacrificed, liver and body weights were recorded, and liver and serum samples were stored at −80°C until use.

### Western blot analysis

Whole liver protein extracts were prepared with RIPA buffer (Sigma, Cat# R0278) containing protease and phosphatase inhibitors (Roche, 1183617011). Protein concentrations and quantification were determined by following the manufacturer’s instructions for the BCA protein assay kit (Thermo Fisher Scientific, Cat# 23225). Band visualization and quantification were performed on SDS-PVDF membranes of 10% Acrylamide gels. Protein of 40 μg was loaded on each well. The following are the detected antibodies. Rabbit anti-Human/Mouse GYS2 (Proteintech, 22371-1-AP), Rabbit anti-Human/Mouse Glycogen synthase [p Ser641] (Novus bio, NBP2-67315), rabbit anti-human/mouse PYGL antibody (Proteintech, 15851-1-AP), rabbit anti-human/mouse Glut2 polyclonal antibody (Proteintech, 20436-1-AP), rabbit anti-human/mouse ADRP/Perilipin 2 Polyclonal antibody (Proteintech, 15294-1-AP), rabbit anti-human/mouse Alpha Smooth Muscle antibody (Novus, NBP1-30894), mice anti-human/mouse AKT antibody (R&D, MAB 2055), mice anti-human/mouse phospho-AKT antibody (R&D, MAB 887), rabbit anti-human/mouse Insulin Receptor-beta antibody (Proteintech, 20433-1-AP), and rabbit anti-human/mouse beta Actin polyclonal antibody (Proteintech, 20536-1-AP).

### RNA isolation and cDNA preparation

Total RNA was extracted from 50 to 100 mg of liver samples using Trizol (TRI) reagent (Bio-Lab, Cat# 009010233100) in which liver sample was homogenized in 1 mL of TRI reagent for 5 min at room temperature using Tissuelyser LT (QIAGEN), followed by the addition of 0.2 mL chloroform (Bio-Lab, Cat#03080521), tightly covered, shacked vigorously for 15 s and allowed to stand for 2–15 min at room temperature. The mixture was centrifuged at 12,000 × *g* for 15 min at 2–8°C. The colorless upper aqueous phase (containing RNA) was transferred to a fresh tube, and 0.5 mL of 2-propanol (Bio Lab, Cat# 16260521) was added, mixed, and allowed to stand for 5–10 min at room temperature, then centrifuged at 12,000 × *g* for 10 min at 2–8°C. The supernatant was removed, and the RNA pellet was washed by adding a minimum of 1 mL of 75% ethanol; the sample was vortexed and then centrifuged at 7,500 × *g* for 5 min at 2–8°C. The RNA pellet was briefly dried for about 5–10 min by air-drying, and then the pellet was re-suspended in RNase-DNase-free water. RNA was quantified using a Nanodrop machine at the central research lab, the Hebrew University of Jerusalem. Samples were stored at −80°C until needed. Complementary DNA (cDNA) was synthesized from 2 μg of total RNA using a High-Capacity cDNA Reverse Transcription Kit with RNase Inhibitor (Applied Biosystems, Cat # 4374966) following the manufacturer’s instructions. Samples were stored at −20°C until needed. RT-PCR was performed for the quantification of the expression of the genes that encoded *alpha-smooth muscle actin* (*αSMA*) (Applied Biosystems, Mm0072512_S1 Acta2, Lot # 1812381) and *Glucose transporter 2* (*Glut2*) (Applied Biosystems, Mm00446229_m1 SLC2a2, Lot # 1790842) compared to *GAPDH* as a housekeeping gene (Applied Biosystems, Mm99999915_g1, Lot # 1703322) by using a TaqMan™ Fast Advanced Master Mix (Applied Biosystems, Cat # 4444964) following the manufacturer’s instructions.

### Serum biochemistry

Mice cardiac blood samples were collected on the day of sacrifice and centrifuged at 5,000 rpm for 15 min at 4°C. Serum ALT (Abcam; ab285263, sensitivity: 4 pg/mL), AST (Abcam; ab263882, sensitivity: 39 pg/mL), and TRG (Abcam; ab65336, sensitivity: > 2 μM) were measured using Enzyme-Linked Immunosorbent Assay (ELISA) kits. All reagents and samples were brought to room temperature (18–25°C) before use. A volume of 100 μL of each standard and sample was added to the appropriate wells and incubated for 2.5 h at room temperature with gentle shaking. The solution was discarded, and the wells were washed four times with 1X wash solution; washing was performed by filling each well with wash buffer (300 μL) using a multichannel pipette or auto-washer. After washing, the liquid was completely removed at each step. A 100 μL of 1× prepared detection antibody was added to each well and incubated for 1 h at room temperature with gentle shaking. One hundred microliters of a prepared streptavidin solution were added to each well and incubated for 45 min at room temperature with gentle shaking. A 100 μL of TMB One-Step Substrate Reagent (Item H) was added to each well and incubated for 30 min at room temperature in the dark with gentle shaking. Finally, 50 μL of Stop Solution (Item I) was added to each well. The absorbance at 450 nm was immediately read using an ELISA reader (Tecan M100 plate reader).

### Serum C-peptide levels

The serum C-peptide 2 level was determined by ELISA using Rat/Mouse C-peptide 2 kit (Merck Millipore, Cat# EZRMCP2-21 K, sensitivity: 15 pM).

### Serum malondialdehyde assay

The serum MDA level was determined by ELISA using MDA assay kit (Abcam, Cat#ab238537).

### Homeostasis model assessment

Homeostasis model assessment (HOMA-IR) is a model of the relationship between glucose and insulin that predicts fasting steady-state glucose (mmol/l) and fasting serum C-peptide (nmol/l). The product of fasting glucose and fasting C-peptide is an index of hepatic insulin resistance. Here, we used a computerized model with a higher accuracy, the HOMA 2 calculator,^1^ to calculate the HOMA-IR score. The formula involves the introduction of data regarding glycemia (mmol/l or mg/dl), insulinemia (pmol/l or μU/mL), or C-peptide (nmol/l or ng/mL), automatically calculating %B, %S, and IR.

### Histological assessments of liver injury

One-third of the posterior liver was fixed with 4% formalin for 24 h at room temperature and then embedded in paraffin in an automated tissue processor (HIS-TSQ; MRC). Microtome Sectioning Tutorial (7 μm; HIS-2268; MRC) was deparaffinized by immersion in xylene. The sections were then rehydrated by passing them through a graded alcohol series, starting with absolute alcohol and ending with distilled water. H&E staining was used to evaluate steatosis, necro-inflammatory regions, and apoptotic bodies as mentioned in Table 1. Masson’s trichrome (MTC, ab150686, Abcam) was used to visualize the connective tissue. A veterinary pathologist assessed all histopathological findings and reported assessment grades. To quantify the fibrotic area, stained slides were scanned using a Zeiss microscope equipped with image analysis software (ImageJ), which was used to outline the fibrotic areas within the tissue section.

**Table 1:** H&E assessment parameters.

Item	Definition	Score/Grade
Steatosis grade	Lower to medium evaluation of parenchymal involvement by steatosis	
	<5%	0
	5–33%	1
	>33–66%	2
	>66%	3
Location	Predominant distribution pattern	
	Zone3	0
	Zone1	1
	Azonal	2
	Panacinar	3
Fibrosis stage	None	0
	Persinusoidal or periportal	1
	Mild, Zone3, persinusoidal	1A
	Moderate, Zone3, persinusoidal	1B
	Portal/ periportal	1C
	Persinusoidal and Portal/ periportal	2
	Bridging fibrosis	3
	Cirrhosis	4
Inflammation		
Lobular inflammation	Overall assessment of all inflammatory foci	
	No foci	0
	<2 foci per 200X field	1
	2–4 foci per 200X field	2
	>4 foci per 200X field	3
Microgranulomas	Small aggregates of macrophages	
	Absent	0
	Present	1
Large lipogranulomas	Usually in portal areas or adjacent to central veins	
	Absent	0
	Present	1
Portal inflammation	Assessed from low magnification	
	None to minimal	0
	Greater than minimal	1
Liver cell injury		
Ballooning	None	0
	Few balloons’ cells	1
	Many cells/prominent ballooning	2
Acidophil bodies	None to rare	0
	Many	1
Pigmented macrophages	None to rare	0
	Many	1
Megamitochondria	None to rare	0
	Many	1

### Statistical analysis

Statistical differences will be analyzed either with the two-tailed unpaired Student’s *t*-test (For comparison between two groups) with one-way or two-way ANOVA with Newman–Keuls’ post-tests among multiple groups using Graph pad Prism 5.0 (GraphPad Software, La Jolla, CA).

## Results

### Total body weights were reduced in RD-fed following D- and L-Glu supplemented and were unchanged in HFD-fed mice

**Figure 1:**
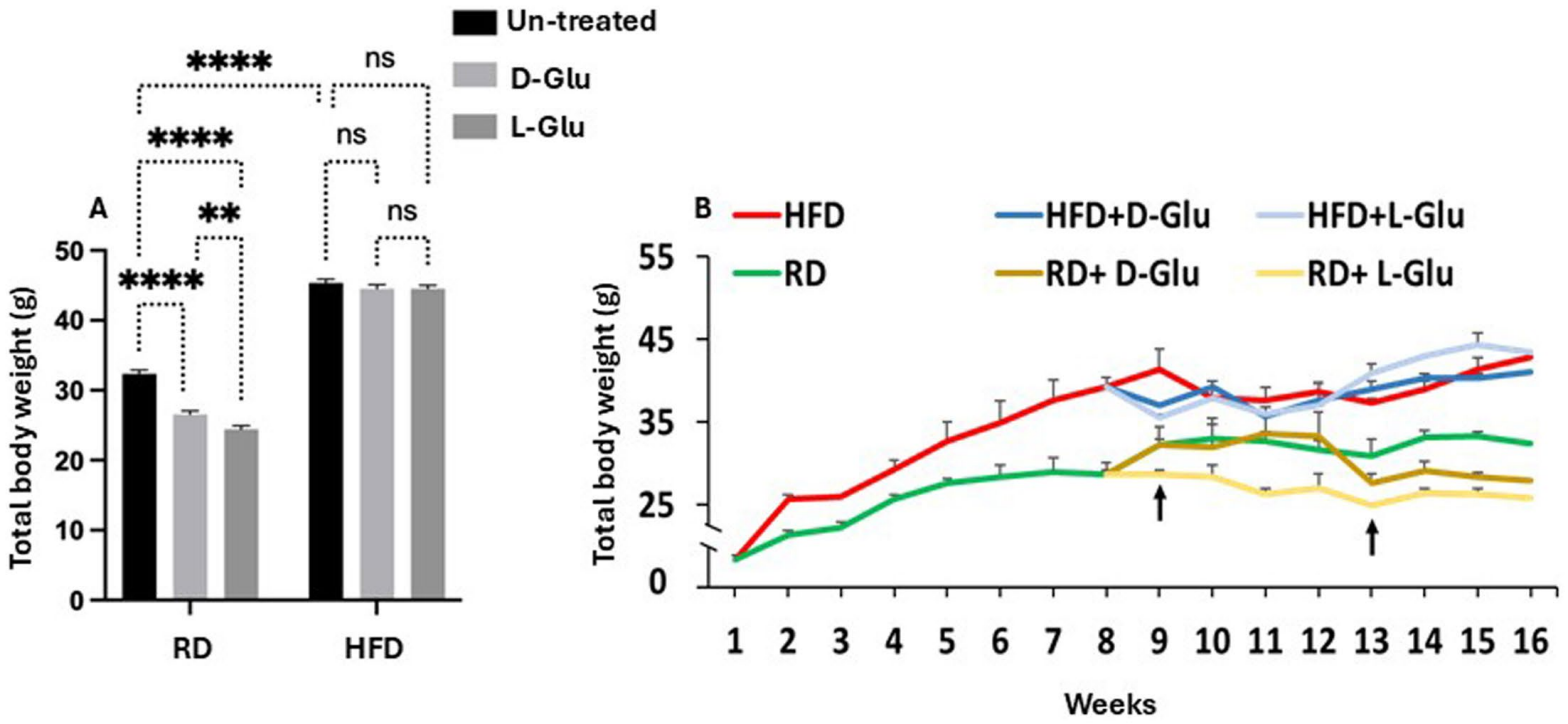
Changes in body weight following glucose supplementation. **(A)** Body weight measurements in grams (g) following D- and L-Glu supplementations in RD and HFD-fed mice groups at week 16. **(B)** Changes in body weight kinetics through 1-week interval readings for 16 weeks. Paired and unpaired Student’s *t*-test and ANOVA were used for statistically significant differences. *p* value was compared between RD and HFD control groups or within RD or HFD groups. *p < 0.05; **p < 0.01; ***p < 0.001.

D- and L-Glu were supplemented to drinking water starting from week 8 of the experiment, as mentioned in the section “Materials and methods.” At week 16 (sacrifice date), HFD-fed mice exhibited a ~ 32.7% increase in body weight (Figure 1A) compared to RD-fed mice. Additional water supplementation of D-Glu to the RD mice groups caused a decrease of 13.55% in their total body weight, while the L-Glu had reduced body weight by about 20% (*p* < 0.002). HFD-fed mice receiving D- and L-Glu supplementations had comparable body weight to the untreated counterparts (P = ns). Figure 1B shows kinetic changes in body weight from week 1 to week 16. In the RD-fed mice group, a reduction in body weight was noticed at week nine following supplementations of L-Glu; this was considered an early effect. However, a reduction in body weight was observed at week 13 in the RD-fed mice following supplementations of D-Glu (late effect) (Figure 1B).

### L-Glu caused increased water consumption and total calories in HFD-fed mice

**Figure 2:**
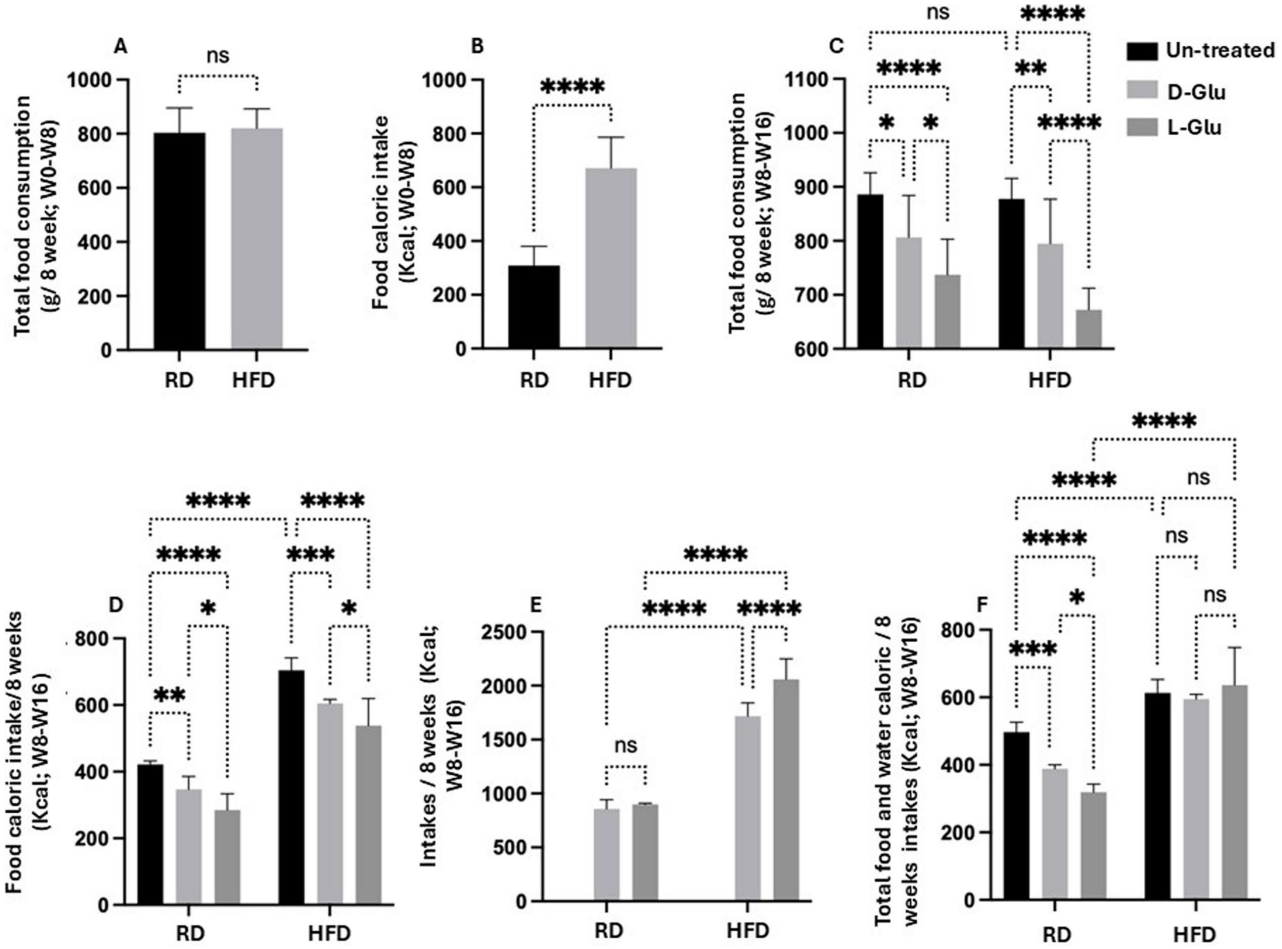
Calculated calories from consumed food and glucose supplementation in drinking water. **(A)** Total food consumption in g/8 weeks from week 0 to week 8 **(B)** Food caloric intakes in Kcal from week 0 to week 8 **(C)** Total food consumption in g/8 weeks from week 8 to week 16. **(D)** Food caloric intakes/8 weeks in Kcal from week 8 to week 16. **(E)** Total water caloric intakes in Kcal from week 8 to week 16. **(F)** Total-food and water-caloric intakes/8 weeks in Kcal from week 8 to week 16. Paired and unpaired Student’s *t*-test and ANOVA were used for statistically significant differences. *p* value was compared between RD and HFD control groups or within RD or HFD groups. *p < 0.05; **p < 0.01; ***p < 0.001; ****p < 0.0001.

To address changes in body weight observed in the RD-fed mice, we calculated total food consumption and diet calories obtained from consumed food and glucose supplementation in drinking water. The total amount of consumed diet and water intake was measured weekly. In addition, total food consumption was calculated before and following supplementations with glucose. Figure 2A summarizes averages of total food consumed calculated by grams before glucose supplementation (week 0 to week 8). Results show no differences in total food consumption between RD and HFD-fed mice groups. In parallel, HFD-fed mice display more calorie intake (1.6-fold; *p* < 0.001) as compared to RD-fed mice (Figure 2B). We also assessed total food consumption and calculated diet calorie intake following glucose supplementation from week 8 to week 16. Figure 2C shows similar food consumption patterns between mice fed with RD compared to HFD (P = ns). Both mice groups receiving the D- and L-Glu supplementations caused a reduction in total food intake to 1.03-fold in all mice groups (*p* < 0.001). These reductions were accompanied by reduced diet calorie intake in RD and HFD-fed mice (Figure 2D). Although RD and HFD-fed mice with no glucose supplementations had comparable food intake, the HFD-fed mice showed high (1.56-fold) total calorie intake. The results also demonstrate that the HFD-fed mice might not need to increase their eating habit because of the high-calorie intake. Consumed water glucose supplementations were also included to better calculate total diet calories. Table 2 shows the amounts of drinking water consumed and glucose supplementations in mice groups in ml volume/week. Figure 2E shows the total calorie intake obtained from water. Results showed an increase in calculated calories from water consumption in mice fed with HFD compared to the RD ones, while RD-fed mice showed similar water intake and water calories. These data were comparable to the expected increase in water consumption observed in Table 3 and showed significant effects in favor of L-Glu in the HFD-fed mice. Figure 2F represents a summary of total calories calculated following food and water consumption in all mice groups. Data indicate reduced total calories in mice fed with RD receiving the D- and L-Glu supplementations in consistent with their reduced total food consumptions (Figure 2B) and same water intake (Table 3), which might explain the reductions in their body weight summarized in Figure 1B and may suggest fluctuations in their diet behavior resulted in loss of appetite. Moreover, the same calorie intake between mice groups HFD-fed mice with or without the Glu supplementations may explain their sustained body weight, as seen in Figure 1B.

**Table 2:** Daily consumed water in ml/week.

Daily drink volume (mL/week)	NT	D-Glu	L-Glu
RD	5	5	5
HFD	9	10	11

**Table 3:** NAS activity score.

	RD	RD + D-Glu	RD + L-Glu	HFD	HFD + D-Glu	HFD + L-Glu
Hepatic ballooning	0	0	0	2	1	0
Lobular inflammation (LI)	0	0	0	1	0	0
Steatosis (S)	0	0	0	3 (63%)	3 (74%)	3 (85%)
NAS scoring	0	0	0	6	4	3

Our results indicated a reduction in food intake in the HFD-fed mice received Glu supplementations, however, high-calorie intake was apparent in mice with the L-Glu supplementation, which drank more water and substituted the reduced calories obtained from food, indicating the significant effects of sweeteners in contributing to total calorie gain.

### HFD-fed mice developed larger livers while significantly reduced following D- and L-Glu supplementations

**Figure 3:**
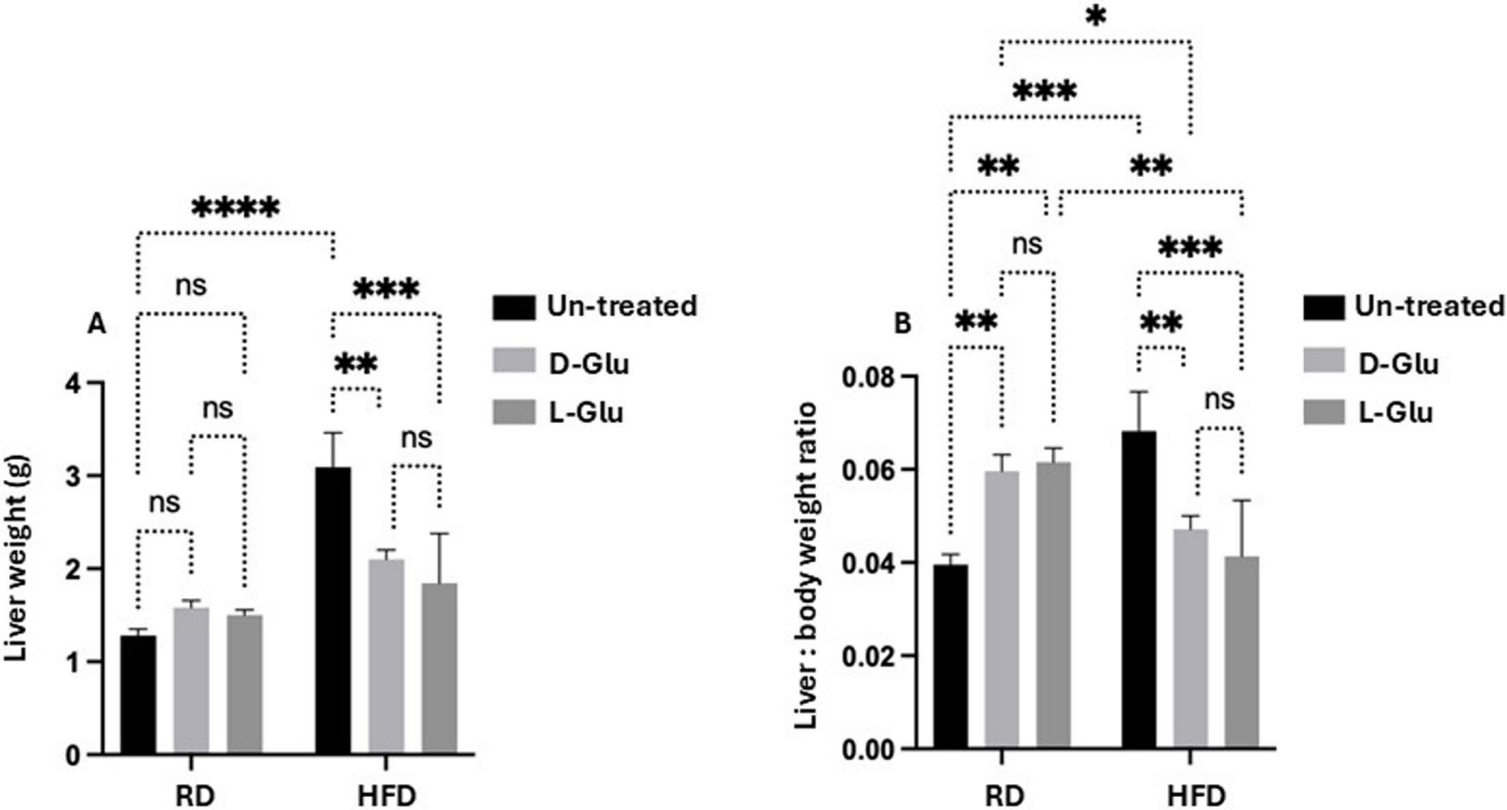
Changes in liver weight. **(A)** The whole mouse’s liver was weighed in week 16, and **(B)** liver to body weight ratio was calculated. Our data indicate that both D- and L-Glu supplementations had; data are presented in grams (g). Paired and unpaired Student’s *t*-test and ANOVA were used for statistically significant differences. The *p* value was compared between the RD and HFD control groups or within the RD or HFD groups. *p < 0.05; **p < 0.01; ***p < 0.001; ****p < 0.0001.

To further characterize our HFD-fed mice model, livers were obtained from all mice groups and weighed. HFD-fed mice with no D- and L-Glu supplementations developed larger livers (Figure 3A) and were significantly heavier than the RD-fed mice by 2.17-fold increase. Liver weights in RD-fed mice showed no changes following the D- and L-Glu supplementations, while fewer liver weights were significantly noticed in the HFD-fed mice group of about 18.2 and 18.1% following the D- and L-Glu supplementations, respectively. The same pattern of data was obtained concerning liver to body weight ratio (Figure 3B). Our data indicate that both D- and L-Glu supplementations had comparable results in achieving lower liver weights in HFD-fed mice.

### Characterization of inflammatory profile in mice fed with RD and HFD following sugar supplementations

**Figure 4:**
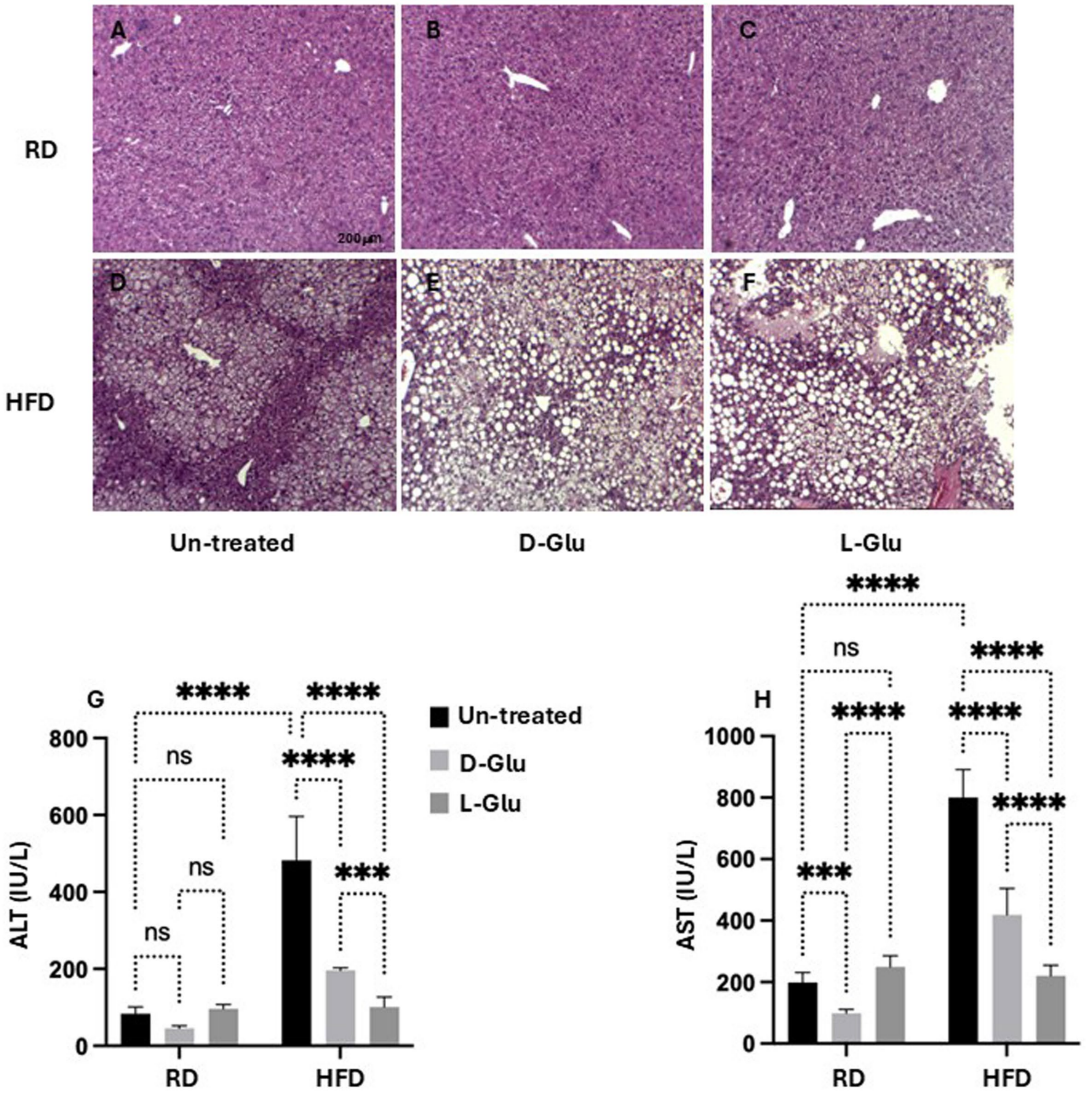
Inflammatory profile assessment: Representative sections of immunohistochemically liver staining with H&E (original magnification 10×) are shown **(A–F)**, scale 200 μM. Liver injury markers of **(G)** serum ALT and **(H)** serum AST were assessed. Paired and unpaired Student’s *t*-test and ANOVA were used for statistically significant differences. *p* value was compared between RD and HFD control groups or within RD or HFD groups. **p < 0.01; ***p < 0.001; ****p < 0.0001.

To better understand alterations in histopathological findings, livers were assessed for inflammation and steatosis by H&E and for histological visualization of collagenous connective tissue fibers by Masson’s trichrome (MTC) staining. In Figure 4A, no histopathological finding was observed on liver tissues obtained from the RD-fed mice. In contrast, microscopic examination of H&E tissue slides revealed higher hepatocyte lipid droplet accumulation in the livers of HFD-fed mice of steatosis grade of 3 with panacinar location and micro-vesicular steatosis (Figure 4D). In addition, livers showed portal inflammation greater than minimal, prominent ballooning, many acidophil bodies, and many mega-mitochondria. D- and L-Glu supplementations did not affect histopathology on livers obtained from mice fed with RD (Figures 4B, C). In parallel, D- and L-Glu supplementations showed increased hepatocyte ballooning (Figure 4E) and steatosis (Figure 4F). Table 3 summarizes the differences in MASLD activity score (NAS) between and among the experimental groups. Data indicate steatosis sub-grading scores in the liver of HFD-fed mice receiving the D-Glu of 74% and L-Glu of 85% compared to 63% in untreated mice. The increase in steatosis grading is well noticed following the L-Glu supplementation in Figure 4F. Biochemical marker outcomes of ALT and AST serum levels were evaluated. Figure 4G shows ALT, and Figure 4H shows AST serum levels with no significant changes in their levels in the mice groups fed with RD following the D- and L-Glu supplementations; results were consistent with the H&E staining. In parallel, both D- and L-Glu supplementations caused a significant reduction in ALT and AST serum levels comparable with the H&E assessments. These data suggest that propagation in liver injury following the L-Glu supplementation in HFD-fed mice resulted in more steatosis and reduced hepatocyte ballooning, which might, in part, indicate accelerated de-novo lipogenesis and, therefore, accumulated lipids in the liver. In addition, reduced ALT/AST serum levels could indicate a chronic liver injury and highlight the issue of the fast uptake rhythm of L-Glu inside the cells, which needs further and future study to confirm this phenomenon.

### Characterization of liver fibrosis profile in RD and HFD-fed mice following sugar supplementations

**Figure 5:**
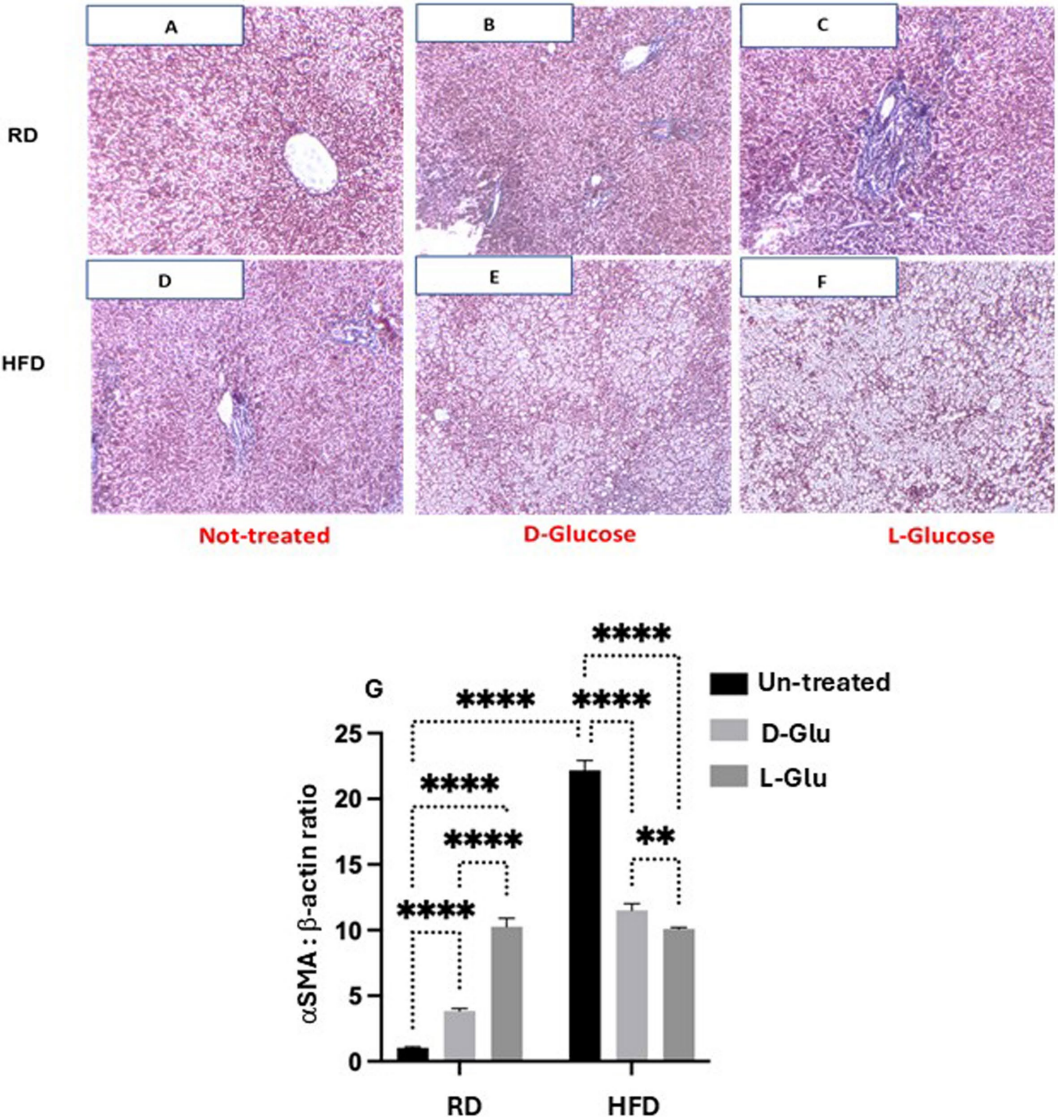
Fibrotic profile assessment: Representative sections of immunohistochemically liver staining with trichrome stain (original magnification 100×) are shown **(A–F)**. Liver fibrosis markers of hepatic αSMA expressions were assessed by **(G)** western blot analysis. For western blot analysis, results are presented as the ratio of αSMA to the housekeeping protein (*β*-actin). Paired and unpaired Student’s *t*-test and ANOVA were used for statistically significant differences. *p* value was compared between RD and HFD control groups or within RD or HFD groups. **p < 0.01; ***p < 0.001; ****p < 0.0001.

Discrepancies in inflammatory profile severities in HFD-fed mice drinking Glu could highlight a more advanced state of liver fibrosis because of Glu supplementation’s continuous (prolonged) insults in drinking water. For this purpose, we aimed to stain liver fibrosis using MTC staining for histological visualization of collagenous connective tissue fibers in liver sections. In Figure 5A, no liver fibrosis was observed in RD-fed mice; in contrast, the HFD-fed mice group (Figure 5D) showed high intensities of MTC staining. D- and L-Glu supplementations in RD-fed mice caused more stained tissues with MTC (Figures 5B,C) in favor of the L-Glu group. Data indicate accelerated fibrogenesis in the mice fed with RD mice following D- and L-Glu supplementations. No difference in liver fibrosis visualization was noticed in the HFD-fed mice group following the D- and L-Glu supplementations (Figures 5E,F); however, accumulation of lipid droplets could be observed as presented in Figures 5E,F. We next quantitate liver αSMA (fibrosis marker) using the western blot analysis [Figure 5G, Supplementary Figure 1 (original gels)] and by the RT-PCR (Supplementary Figure 2). Figure 5G shows significant elevations in *αSMA* in the HFD-fed mice (16.4-fold increase; *p* = 0.00005) compared to RD-fed mice. Both D- and L-Glu supplementations significantly induced 2.5-fold and 6.5-fold elevations in αSMA, respectively. In the HFD-fed mice group, both D- and L-Glu supplementations repress αSMA to nearly 1.5-fold compared to the untreated group (*p* = 0.002). These results were also confirmed using the RT-PCR with the same achieved patterns (Supplementary Figure 2). The data obtained from the HFD-fed mice could suggest (1) amelioration in liver fibrosis or (2) severe disruption in liver histology leading to clearance of fibrosis in liver sections. Therefore, several experiments were conducted to clarify the effects of glucose on regulating liver injury profiles.

### Reduce hepatic ADPR expression in the HFD-fed mice receiving the D-and L-Glu supplementations, indicating less hepatic lipid uptake

**Figure 6:**
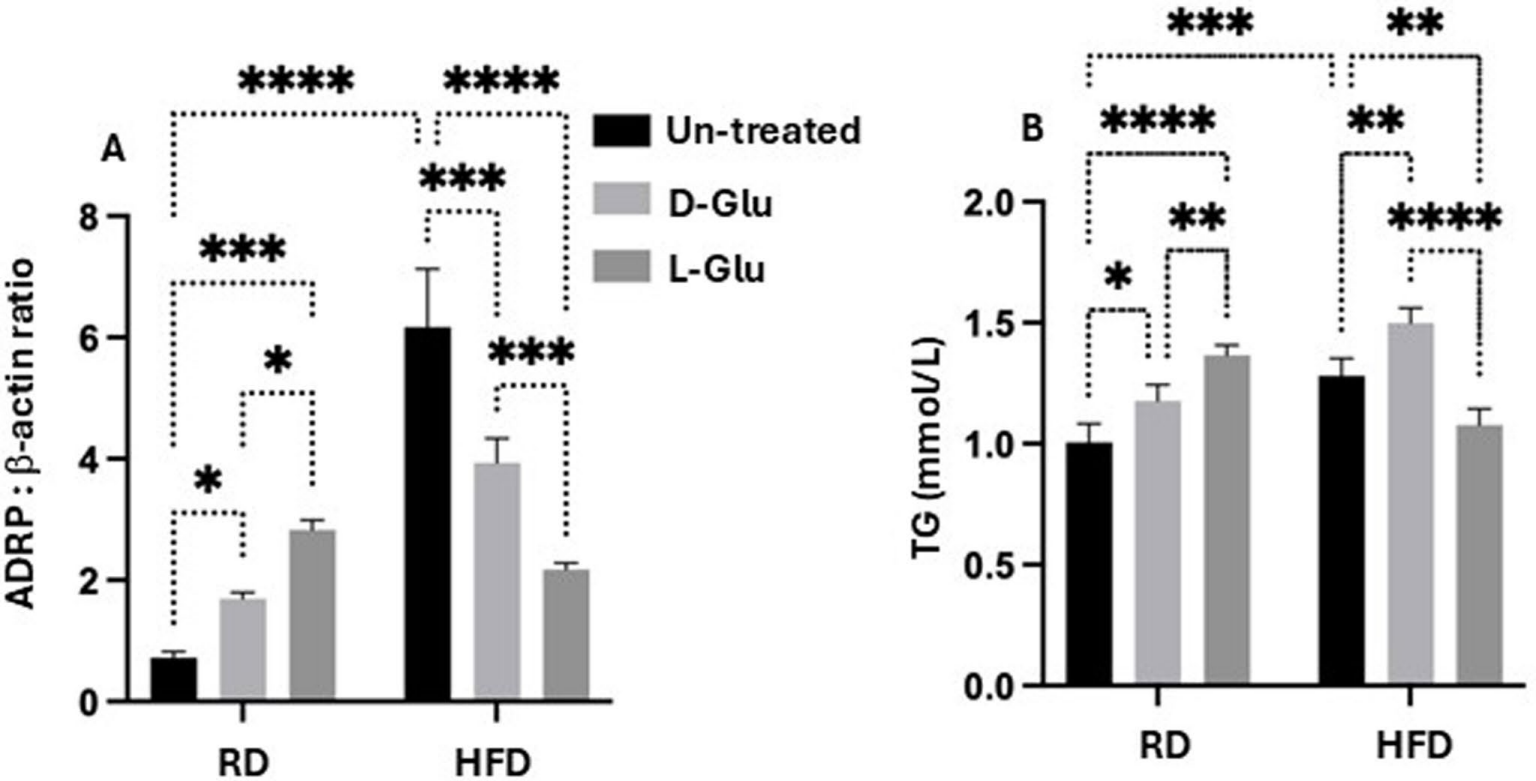
Lipid profile assessment: **(A)** Adipose differentiation related-protein (ADRP) was assessed by western blot analysis, and data were represented by the ratio of ADRP to the housekeeping protein (β-actin). **(B)** Serum triglyceride (TG mmol/L) levels were assessed. Paired and unpaired Student’s *t*-test and ANOVA were used for statistically significant differences. The *p* value was compared between the RD and HFD control groups or within the RD or HFD groups. *p < 0.05; **p < 0.01; ***p < 0.001; ****p < 0.0001.

The overall data indicates several clinical outcomes from glucose supplementations in the RD and HFD-fed mice groups. While D- and L-Glu supplementations showed a pro-fibrotic effect in the RD-fed mice model, both supplementations caused a reduction in inflammatory as well as fibrotic markers in their HFD-fed counterparts. These data were accompanied by reduced liver weights in the HFD-fed mice model. Therefore, we next asked whether the decrease in liver weight observed in the HFD-fed mice following glucose supplementation could result from less accumulation in fat deposits. For this purpose, we evaluated expressions of adipose differentiation-related protein (ADRP), which facilitates the uptake of long-chain fatty acids and the formation of lipid droplets in lipid-accumulating cells in hepatocytes (12). It has been shown that decreased expression of ADRP decreases the fatty liver while increasing its expression is associated with several metabolic diseases like type 2 diabetes, insulin resistance, and heart diseases. Figure 6A (Supplementary Figure 1, original gels) shows elevated ADRP expressions in HFD-fed mice (6.8-fold, *p* = 0.003). Supplementation of D- and L-Glu to the RD-fed mice caused elevated expressions of ADRP, which may indicate higher fat uptake and an active lipogenesis process. In contrast, D- and L-Glu supplementations to the HFD-fed mice caused a significant reduction in ADRP expressions to 1.3 and 2.8-fold, respectively. While these results could suggest less lipid uptake in the HFD-fed mice, we hypothesized that lipids could accumulate outside the livers. For this reason, serum triglycerides were evaluated. Figure 6B shows serum TG elevated in the HFD-fed mice at 1.2 mmol/L compared to 1.06 mmol/L in the RD-fed mice (*p* < 0.05). These results indicate more lipid uptake and accumulation in the HFD-fed mice. Following the Glu supplementations, the same high lipid uptake and accumulation patterns were obtained in the RD-fed mice (1.15 mmol/L in the D-Glu and 1.34 mmol/L in the L-Glu).

In the HFD-fed mice, further elevations in serum TG were observed only following the D-Glu (1.6 mmol/L; *p* = 0.0003), however were reduced following the L-Glu supplementations to 1.2 mmol/L; *p* = 0.0003. The results obtained so far could indicate the following: 1-In the RD-fed mice group, serum levels of TG following glucose supplementations were elevated, together with an increase in their hepatic uptake, indicating normal hemostasis of lipid trafficking (efflux) and the liver’s ability to deal with additional sugar intake (later converted to fat; known as de-novo lipogenesis (DNL)). 2-Reduced hepatic ADPR expressions were noticed in the HFD-fed mice group receiving the D-Glu, which was in line with its accumulation in the blood, indicating less hepatic lipid uptake. 3- Although hepatic ADRP expressions were also reduced in the HFD-fed mice group receiving the L-Glu, a significant decrease in serum TG levels was noticed, suggesting less accumulation of lipids in the blood and raising the issue of lipid accumulation in other organs or/and increase in clearance of lipid through lipid peroxidation as a result diffusion of lipids. The above data speculates that reduced fat storage in HFD-fed mice could partly explain the reduced liver weight observed in the HFD-fed mice following the glucose supplementations.

### Glucose induces lipid peroxidation in both RD and the HFD-fed mice

**Figure 7:**
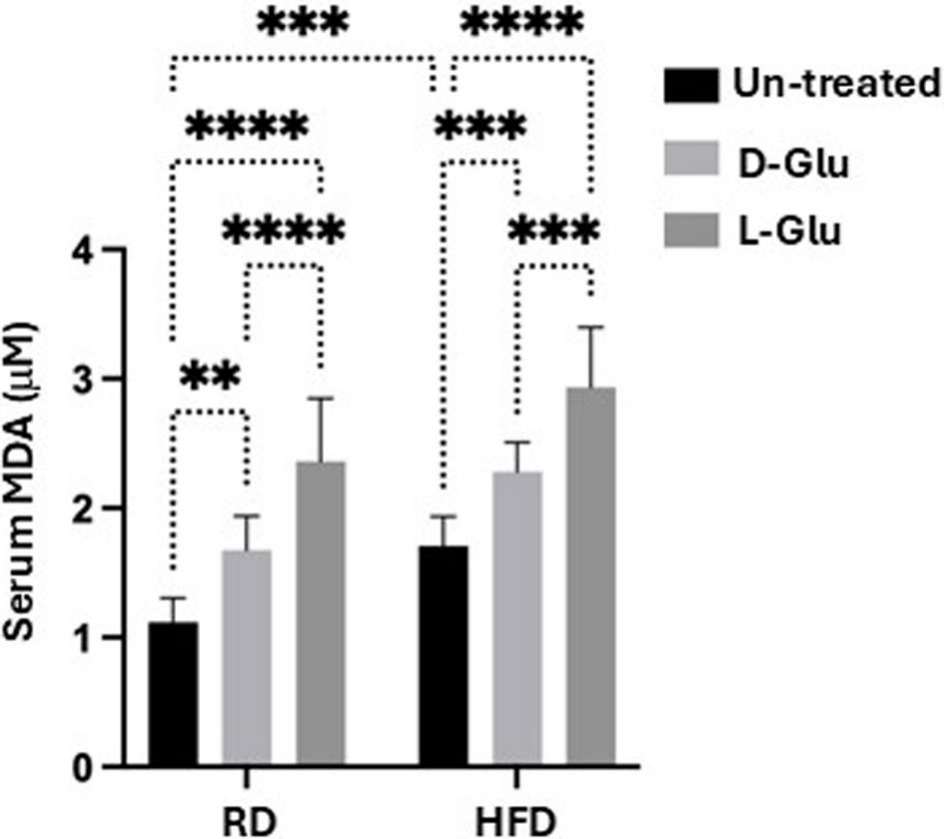
Serum lipid peroxidation: Malondialdehyde (MDA) was used as a marker of lipid peroxidation. Results show serum MDA levels as evaluated by ELISA. Paired and unpaired Student’s *t*-test and ANOVA were used for statistically significant differences. *p* value was compared between RD and HFD control groups or within RD or HFD groups. **p < 0.01; ***p < 0.001; ****p < 0.0001.

To explain the less lipid uptake and less serum TG in the HFD-fed mice receiving the L-Glu, we assessed lipid peroxidation, hepatic glucose transporter expression, and insulin serum levels. We have checked for malondialdehyde (MDA), a product of oxidative degradation of lipids. Several studies showed oxidative stress to be implicated in the pathogenesis of type 2 diabetes and its complications. Metabolic disturbances contribute to oxidative stress and compromise the antioxidant defense system in type 2 diabetes patients (13). Figure 7 shows high serum MDA levels in HFD-fed mice compared to the RD-fed mice (1.7-fold, *p* < 0.0001). In the RD-fed mice, D- and L-Glu caused significant elevation in serum MDA to 1.14 and 1.21 –folds, respectively (*p* < 0.0001). The same patterns were obtained in the HFD-fed mice, showing further elevated serum MDA levels following the glucose supplementations (p < 0.0001). These data suggested the direct effects of glucose on accelerating lipid peroxidation in both RD and HFD-fed mice, which was in favor of the latter. The high extent of lipid peroxidation in HFD-fed mice could result from inhibited expressions of ARDP. In the RD-fed mice, it could be suggested due to the increase in de-novo lipogenesis (RD contains a high-carbohydrate diet).

### Decreased in Glut2 transporter in HFD-fed mice

**Figure 8:**
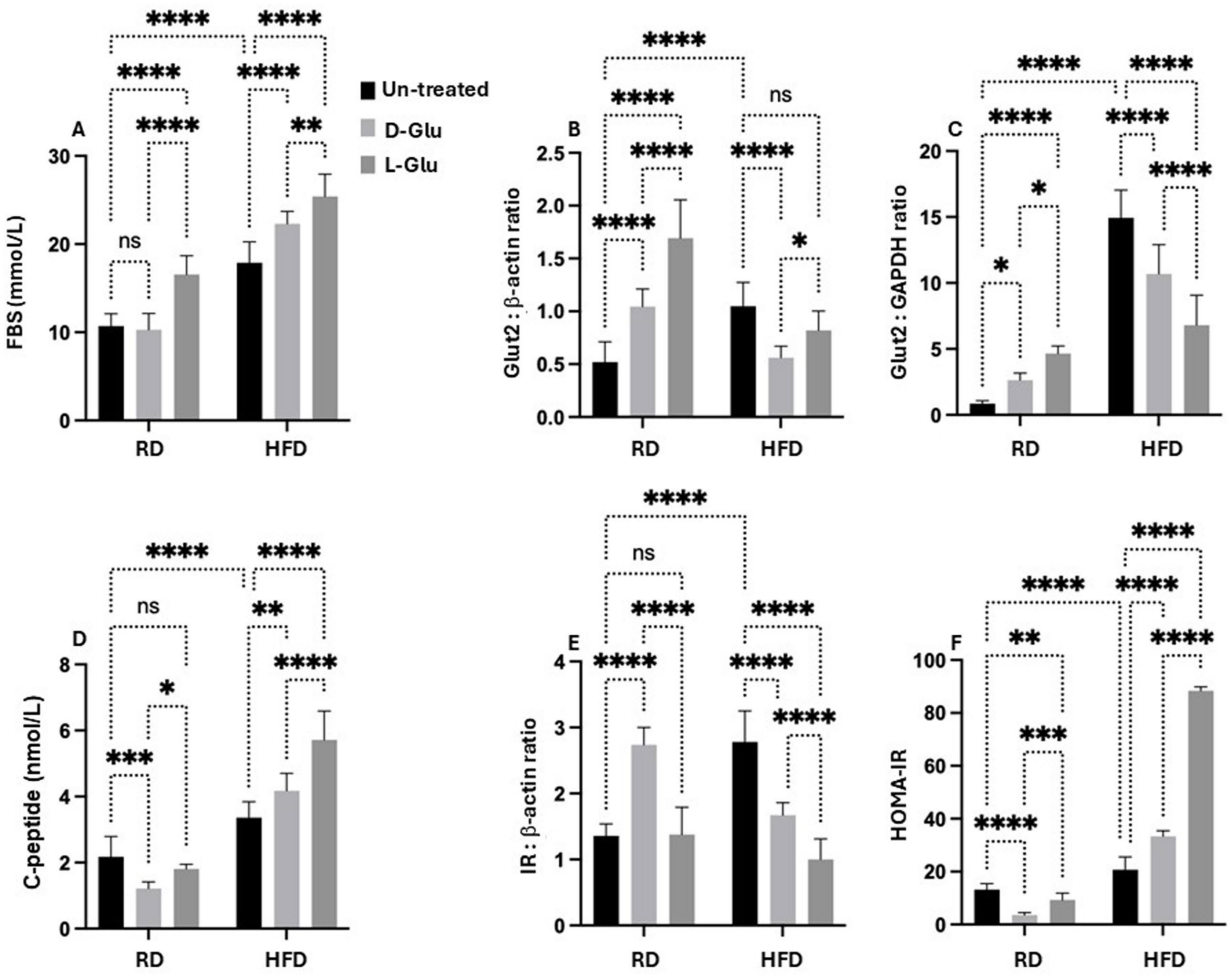
Metabolic profile assessment: **(A)** Fasting blood sugar (FBS) was assessed as indicated in materials and methods. *GLUT2* expressions were quantitated by **(B)** western blot and **(C)** RT-PCR methods. **(D)** Serum insulin C-peptide measured by ELISA. **(E)** Hepatic expression of inulin receptor (IR) was evaluated from liver sections by western blot **(F)** HOMA2 calculator calculated HOMA-IR. Paired and unpaired Student’s *t*-test and ANOVA were used for statistically significant differences. *p* value was compared between RD and HFD control groups or within RD or HFD groups. *p < 0.05; **p < 0.01; ***p < 0.001; ****p < 0.0001.

To further assess the metabolic assessments of glucose on lipid peroxidation, we evaluate fasting blood sugar (FBS) in our mice model. In hepatocytes, glucose can be stored as glycogen, degraded through the glycolytic pathway, or converted to fatty acids by the lipogenic pathway. The release of glucose in circulation follows the degradation of glycogen or gluconeogenesis. Glucose also modifies cellular metabolism by allosteric and transcriptional regulation. HFD-fed mice showed high FBS levels (19.2 mmol/L) compared to the RD-fed mice (13.1 mmol/L; *p* = 0.001, Figure 8A). While D-Glu had no significant effects on FBS in the RD-fed mice, L-Glu significantly elevated FBS to 1.1–fold (*p* = 0.02). D- and L-Glu supplementations increased FBS in the HFD-fed groups to 1.2-fold in both groups (*p* = 0.01). These results indicate that glucose supplementation and high FBS insults could contribute to lipid peroxidation, as shown in Figure 7. Hyperglycemia obtained in our HFD-fed mice following glucose supplementations could indicate less hepatic glucose uptake and point to modulation in hepatic glucose transporter in these mice. GLUT2 is the major glucose transporter of hepatocytes in rodents and humans (44) The generally accepted role of this transporter is to take up glucose during the absorptive phase and to release it in the blood during fasting (14). We evaluated hepatic *GLUT2* expressions by the western blot and RT-PCR methods. Figure 8B (Supplementary Figure 1, original gels) indicates high expressions of GLUT2 in the HFD-fed mice compared to the RD-fed mice (1.2-fold, *p* = 0.001). D- and L-Glu supplementations, while inducing elevated expressions of *GLUT2* in the RD-fed mice, significantly inhibited *GLUT2* expressions in the HFD-fed mice groups (*p* < 0.01). The RT-PCR analysis achieved the same pattern of results (Figure 8C). Our results indicate that adding sugar to the existing high-carbohydrate diet (70% Kcal) in the RD-fed mice stimulates GLUT2 expression. HFD-fed mice [weaning on a high-carbohydrate diet (20% Kcal)], while having elevated GLUT2 expressions, most probably to uptake more glucose, were unexpectedly shown to be inhibited when the HFD-fed mice were combined with additional sugar supplementations.

### Hyperinsulinemia in HFD-fed mice is correlated with elevated serum glucose

Although the carbohydrate content in the HFD-fed mice, even with the addition of sugar supplementations, did not reach the RD content with no expected elevation in the GLUT2, this could point to an inhibitory pathway that led to GLUT2 downregulation. In this aspect, we sought to evaluate whether insulin could induce reductions in GLUT2. Hepatic GLUT2 is not pathologically involved in states of glucose intolerance. Therefore, serum insulin C-peptide levels were evaluated as a marker of insulin production rate and hepatic insulin receptors (15). Figure 8D shows a significantly higher serum insulin C-peptide level in the HFD-fed mice compared to the RD-fed mice 1.6-fold (*p* < 0.001). In the RD-fed mice, D- and L-Glu supplementations induce a reduction in serum insulin C-peptide, reflecting a state of hypo-insulinemia. These results were associated with elevations in the hepatic insulin receptor [Figure 8E, Supplementary Figure 1 (original gels)] to 1.67-fold and 1.2-fold in the D- and L-Glu supplemented groups, respectively (*p* < 0.05). This data, together with the elevation in the GLUT2 observed in Figures 8B,C, suggests normal glucose hemostasis due to the normal response of the liver to elevated concentrations of sugar and are in less need of insulin interference. In contrast, in the HFD-fed mice, further increases in the serum insulin C-peptide levels (Hyperinsulinemia) were observed following glucose supplementation accompanied by a reduction in the expression of hepatic insulin receptor (Figure 8E) and inhibited GLUT2 seen in Figures 8B,C suggested a state of insulin resistance. Figure 8F summarizes HOMA–IR score as summarized in the section “Materials and methods.” HFD-fed groups receiving the D- and L-Glu supplementations showed high HOMA-IR to 1.3-fold and 3.4-fold, respectively (*p* < 0.001).

### Glucose flux balance in HFD-fed mice with Glu supplementation

**Figure 9:**
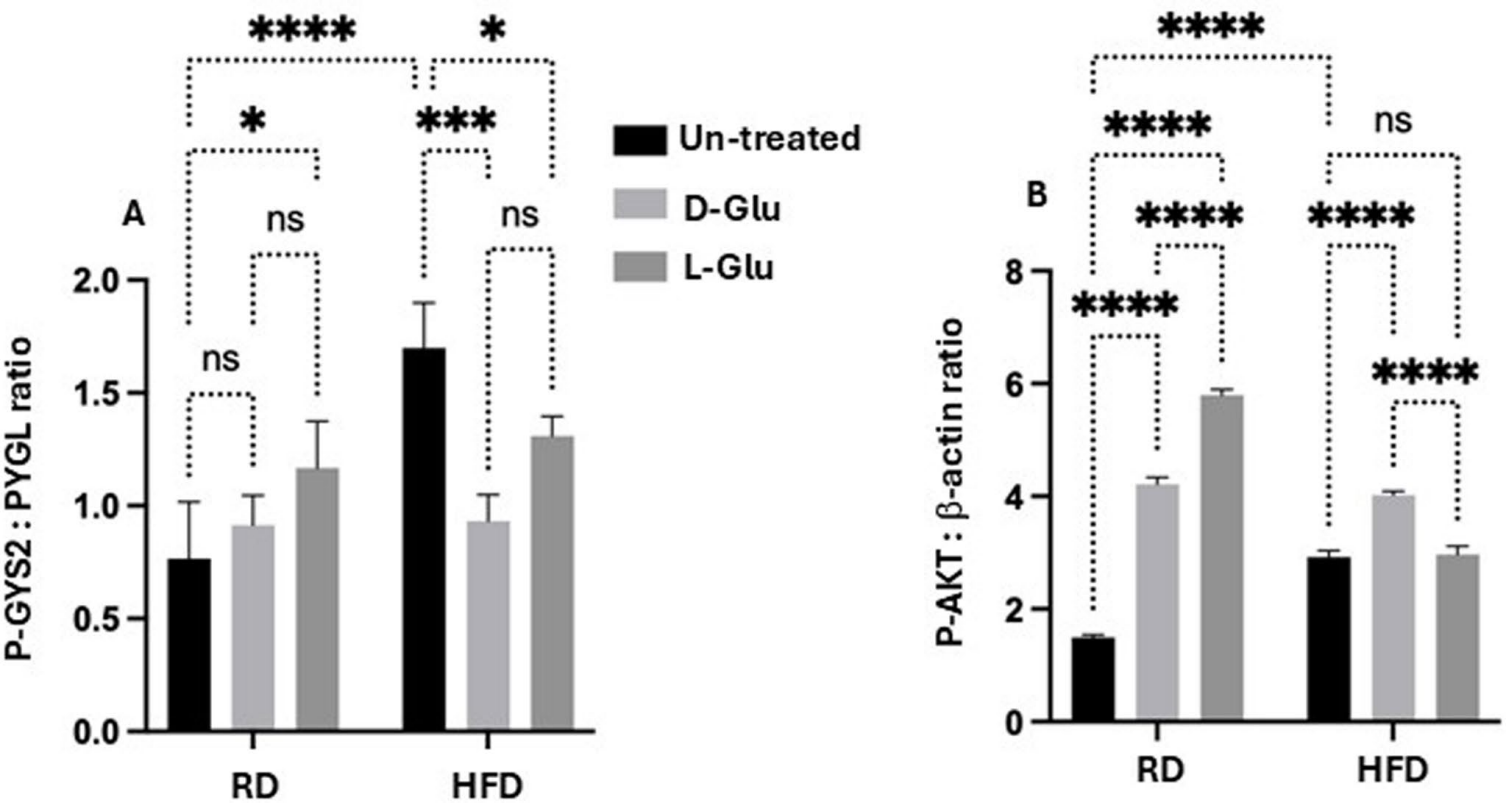
Glucose flux balance regulation and hepatic survival: **(A)** phosphor-glycogen synthase (p-GYS2) to Glycogen degradation (PYGL) ratio was assessed by western blot. A decrease in the ratio indicates a decrease in glycogen degradation. **(B)** Hepatic phosphorylated Akt was quantitated through western blot analysis. Paired and unpaired Student’s *t*-test and ANOVA were used for statistically significant differences. The *p* value was compared between the RD and HFD control groups or within the RD or HFD groups. *p < 0.05; ***p < 0.001; ****p < 0.0001.

To evaluate whether changes observed in the metabolic profile alter glucose flux and, as a consequence, may affect hepatocytes viability, we assessed the enzymes necessary for glucose storage (GYS2; a Glycogen synthase is a key enzyme in glycogenesis, the conversion of glucose into glycogen) as well as for glycogen degradation (PYGL; a liver glycogen phosphorylase, catalyzes the phosphorolysis of an *α*-1, 4-glycosidic bond in glycogen to yield glucose 1-phosphate; glycogen degradation) (16). Moreover, cell survival and metabolism (p-Akt) pathways were evaluated. Figure 9A (Supplementary Figure 1, original gels) shows the ratio of p-GYS2/PYGL assessed by the western blot analysis. Figure 9A indicates that RD-fed mice receiving the D-Glu had a similar ratio to the untreated mice, suggesting the balance of glucose storage and glucose degradation and demonstrate normal glucose hemostasis. In contrast, RD-fed mice receiving the L-Glu had reduced glycogen degradation in favor of glucose storage, indicating high glucose flux [high L-Glu uptake (increase in GLUT R)]. In parallel, both D-and L-Glu supplementations caused an increased glycogen degradation in the HFD-fed mice group (represented as a low p-GYS2/PYGL ratio); results may indicate the demand of the cells to maintain normal levels of the intracellular glucose and suggest less glucose uptake into the cell. These results also align with the high HOMA scores in these two groups. We next evaluated p-Akt as it is being suggested as a mediator for insulin activity (17). p-Akt disturbance, on the other hand, may cause insulin resistance (17). Therefore, we quantitated p-Akt expression in livers obtained from our mice model. Figure 9B (Supplementary Figure 1, original gels) shows elevated expressions of hepatic p-Akt following the D- and L-Glu supplementations in the RD-fed mice. D- and L-Glu supplementations in the HFD-fed mice caused no changes in the liver p-Akt expressions, indicating less responsiveness of the cells to the metabolic stimulation needed for cell survival and viability. The above results may conclude that the livers of HFD-fed mice receiving an extra diet of sugars may affect cell phenotype viability through inhibited p-Akt expressions. Indeed, insulin has been suggested to activate GYS2 through p-Akt (17) and in insulin resistance state, this process could be diminished.

## Discussion

Overconsumption of diet rich in fat and sugar-sweetened beverages are risk factors for developing obesity, insulin resistance and fatty liver disease (18). Some epidemiological studies have shown that artificial sweeteners are beneficial for weight loss and for those who suffer from glucose intolerance and type 2 diabetes mellitus (19). However, accumulating evidence in recent years suggests that artificial sweetener consumption could perturb human metabolism, especially glucose regulation (20, 21). Artificial sweeteners have been found to cause glucose intolerance and induce metabolic syndrome and are associated with higher body weight gain (21). These findings suggest that artificial sweeteners may increase the risk of obesity. However, the specific mechanism through which artificial sweeteners dysregulate the host metabolism remains elusive.

Artificial sweeteners are marketed as a healthy alternative to sugar and as a tool for weight loss, however, the evidence that they are helpful over a longer period is limited (20). By dissociating sweetness from calories, NNS could interfere with physiological responses that control homeostasis (20). Second, by changing the intestinal environment, NNS could affect microbiota and in turn trigger inflammatory processes that are associated with metabolic disorders (20). Third, by interacting with novel sweet-taste receptors discovered in the gut, NNS could affect glucose absorptive capacity and glucose homeostasis (20). Up to date, five NNS (acesulfame potassium, aspartame, neotame, saccharin, and sucralose) are approved by the US food and drug administration (FDA) (20). For instance, Acesulfame potassium (Ace-K), an artificial sweetener, is found to be 200 times sweeter than sucrose (common sugar), present in used soft drinks, drink mixes, frozen desserts, baked goods, candy, gum, and tabletop sweeteners, and in athlete’s protein shake. Although it is considered safe by the FDA, still there are debates about their long-term use and doses (22). Indeed, data suggests that the intended effects of artificial sweeteners do not correlate with what is seen in clinical practice (22).

From this concept, we aimed to study effects of L-Glu (artificial synthesized sugar) intake on animal model of HFD-fed mice. This hypothesis is derived from the idea that obese patients could use artificial sweeteners in an attempt to control their weight and maintain a diet with low calorie. No previous studies were conducted to assess differences in D- and L-Glu intake in patients with obesity and/or with MASLD. For this reason, we adapted the mice model of HFD-fed mice to study effects of D- and L-Glu supplementations on glucose and lipid homeostasis. Moreover, liver profile was also assessed following glucose supplementations.

Our research has concluded some evidence on how glucose supplementations could alter health and disease outcome status. Pre-existing insulin resistance in HFD-fed mice with additional sugar intake caused them to develop a more severe HOMA-IR score (hyperglycemia and hyperinsulinemia), consistent with elevated serum triglyceride levels. Surprisingly, L-Glu induced a more severe HOMA-IR score than D-Glu. Moreover, the hepatic expression of ADRP was suppressed in the mice group receiving the L-Glu, indicating less formation of lipid droplets in lipid-accumulating cells in hepatocytes. This data indicates that the lipid content in the cells regulates ADRP, and its inhibitions are attributed to a preexisting increase in lipid accumulation in the liver. These results were compatible with increased lipid peroxidation following L-Glu intake. Studies showed that fructose-sweetened beverages consumed by human subjects for several weeks resulted in increased hepatic lipogenesis, accumulation of intra-abdominal fat, production of atherogenic lipids, and a marked reduction in insulin sensitivity compared with an isocaloric consumption of glucose (9). The overload of fatty acids and lipid accumulation in MASLD may mechanistically inhibit the synthesis of de-novo fatty acids. Subsequently, the lipid oxidation pathways dominate and induce the net results of lipid clearance (23). An increase in lipid peroxidation following L-Glu supplementation could partly explain reduced liver weights obtained in HFD-fed mice (Figure 3).

Glucose induces oxidative stress and contributes to the inflammatory pathways associated with diabetes and atherosclerosis pathophysiology (24). Specifically, oxidative stress contributes to insulin resistance through an “oxidative-inflammatory cascade (OIC).” Glucose, obesity, and oxidative stress reduce intracellular antioxidant defense mechanisms while activating inflammatory responses from transcription factors and kinases, such as c-Jun N-terminal kinase (JNK), protein kinase C (PKC), and inhibitor of kappa B kinase-*β* (IKKβ) (25). Moreover, some inflammatory pathways, such as activation of IKKβ, have a causative role in the harmful effects of high glucose on endothelial cell function (26).

Non-Nutritive Sugars has been shown to play a role in the pathways regulating glucose absorption from the intestinal lumen into enterocytes in the gut (27). Data obtained in rodents suggest that intestinal sweet taste receptors control both active glucose absorption by modulating expression of sodium-dependent glucose transporter isoform 1 (SGLT) and passive glucose absorption by modulating apical GLUT2 insertion to the intestine (27). No available data on NNS effects on hepatic GLUT2 expressions were studied previously. GLUT2 is the major glucose transporter of hepatocytes in rodents and humans. The generally accepted role of this transporter is to take up glucose during the absorptive phase and release it in the blood during fasting. Hepatic expression on the role of GLUT2 in HFD-fed mice and its modulatory signaling pathway is not well understood. Our data showed elevated hepatic expressions of GLUT2 in HFD-fed mice compared to the RD-fed mice. While D- and L-Glu supplementations induced elevated expressions of GLUT2 in the RD mice, they significantly inhibited GLUT2 expressions in the HFD-fed mice. Our results indicate that additional sugar supplementations to the existing high carbohydrate diet (70% Kcal) in the RD mice stimulate GLUT2 expression. HFD-fed mice weaned on a high carbohydrate diet (20% Kcal) while having elevated GLUT2 expressions, most probably in an attempt to uptake more glucose, have shown to be unexpectedly inhibited when the HFD-fed was combined with additional sugar supplements. Although total sugar content in the HFD-fed mice was less than their RD-fed counterparts, even though they were supplemented with sugar, this did not induce the expected elevation in the GLUT2 and pointed to inhibitory pathways leading to GLUT2 downregulation (elevated insulin). Activation of Akt is the integral result of multiple inputs to regulate hepatic glucose and lipid metabolism. Indeed, studies have shown that insulin regulates GLUT2 expression through phosphorylation of Akt (28). A survey by Rathinam et al. (29); concludes a possible link between Akt activation and GLUT2 synthesis and translocation. In obesity, insulin resistance increases GLUT2 levels, which may further exacerbate metabolic dysfunction in MASLD. However, excess glucose led to internalized GLUT-2 and the insulin receptor together into endosomes in response to insulin (30).

Our data showed a lack of hepatic insulin receptors and GLUT2 in the glucose-supplemented HFD-fed mice, associated with HOMA-IR scores and low p-Akt signaling pathways. A significant association was demonstrated between NNS consumption and obesity in a meta-analysis conducted by Ruanpeng et al. (8). According to a review study by Pearlman et al. (31) in both animal models and humans, NNS may change the host microbiome, leading to decreased satiety, alteration in glucose homeostasis, increased calorie intake, weight gain, and metabolic syndrome. There is no clear evidence of the direct link between NNS and liver injury. Most studies focused on NNS’s potential role in microbiota alteration and dysbiosis and consequently contribute to the progression of MASLD. Dietary factors and increased plasma fatty acid levels may be due to increased triglyceride synthesis, lipolysis, and DNL in the liver, which induce liver injury and fibrosis (32). It has been shown that hepatic steatosis is correlated with the progression of fibrosis (33). Although the mechanism is not fully understood, lipotoxicity induced by severe macro-vesicular steatosis may result in chronic inflammation and oxidative stress, leading to the activation of hepatic stellate cells (34). A high-carbohydrate diet can prime the DNL pathway with a large substrate load and increase rates of DNL importantly this leads to an accumulation of DNL products, fatty acyl chains linked to coenzyme A, which can be incorporated into a plethora of lipid species (9). These lipids may then have further metabolic functions, which may be deleterious in cases of elevated DNL.

Our data indicate the development of liver fibrosis in mice fed with RD (High carbohydrate diet) with additional sugar supplementations, underlining a state of increased rate of DNL, which could partly explain liver fibrosis. In contrast, HFD-fed mice (already with a pre-existing high lipid profile) supplemented with additional sugar intake showed less progression of hepatic fibrosis, most probable because of reduced de-novo fatty acids synthesis. Subsequently, the lipid oxidation pathways become dominant and induce the net results of lipid clearance.

## Conclusion

Individuals widely use non-nutritive sweeteners (NNS) in attempts to lower their overall daily caloric intake, lose weight, and sustain a healthy diet. In our current study, we showed evidence in linking NNS and their implications on the development of metabolic syndrome. The study has suggested that NNS may contribute to the development or worsening of metabolic diseases, including metabolic syndrome, obesity associated with elevated oxidative stress and development of insulin resistance in particularly in mice receiving the HFD with pre-existing high calorie intake and high food consumption.

## Data Availability

The datasets presented in this study can be found in online repositories. The names of the repository/repositories and accession number(s) can be found in the article/Supplementary material.

## Footnotes

^1^ https://www.dtu.ox.ac.uk/homacalculator/download.php

## References

[1] Teng ML Ng CH Huang DQ Chan KE Tan DJ Lim WH. Global incidence and prevalence of nonalcoholic fatty liver disease. Clin Mol Hepatol. (2023) 29:S32–42. doi: 10.3350/cmh.2022.0365, PMID: 36517002 PMC10029957

[2] Vancells Lujan P Viñas Esmel E Sacanella ME. Overview of non-alcoholic fatty liver disease (NAFLD) and the role of sugary food consumption and other dietary components in its development. Nutrients. (2021) 13:1442. doi: 10.3390/nu13051442, PMID: 33923255 PMC8145877

[3] Ling Z Zhang C He J Ouyang F Qiu D Li L. Association of Healthy Lifestyles with non-alcoholic fatty liver disease: a prospective cohort study in Chinese government employees. Nutrients. (2023) 15:604. doi: 10.3390/nu15030604, PMID: 36771311 PMC9921275

[4] Basaranoglu M Basaranoglu G Bugianesi E. Carbohydrate intake and nonalcoholic fatty liver disease: fructose as a weapon of mass destruction. Hepatob Surg Nutr. (2015) 4:109–16. doi: 10.3978/j.issn.2304-3881.2014.11.05, PMID: 26005677 PMC4405421

[5] Gurung P Zubair M Jialal I. Plasma glucose In: StatPearls. Treasure Island, FL: StatPearls Publishing. (2024). Available at: https://www.ncbi.nlm.nih.gov/books/NBK541081/31082125

[6] Pereira RM Botezelli JD da Cruz Rodrigues KC Mekary R Cintra D Pauli J. Fructose consumption in the development of obesity and the effects of different physical exercise on the hepatic metabolism. Nutrients. (2017) 9:405. doi: 10.3390/nu9040405, PMID: 28425939 PMC5409744

[7] Anastasiou IA Eleftheriadou I Tentolouris A Mourouzis I Pantos C Tentolouris N. The use of L-glucose in Cancer diagnosis: results from in vitro and in vivo studies. Curr Med Chem. (2021) 28:6110–22. doi: 10.2174/0929867328666210311112240, PMID: 33719949

[8] Ruanpeng D Thongprayoon C Cheungpasitporn W Harindhanavudhi T. Sugar and artificially sweetened beverages linked to obesity: a systematic review and meta-analysis. QJM. (2017) 110:513–20. doi: 10.1093/qjmed/hcx068, PMID: 28402535

[9] Sanders FW Griffin JL. De novo lipogenesis in the liver in health and disease: more than just a shunting yard for glucose. Biol Rev Camb Philos Soc. (2016) 91:452–68. doi: 10.1111/brv.12178, PMID: 25740151 PMC4832395

[10] Ipsen DH Lykkesfeldt J Tveden-Nyborg P. Molecular mechanisms of hepatic lipid accumulation in non-alcoholic fatty liver disease. Cell Mol Life Sci. (2018) 75:3313–27. doi: 10.1007/s00018-018-2860-6, PMID: 29936596 PMC6105174

[11] Dharmalingam M Yamasandhi PG. Nonalcoholic fatty liver disease and type 2 diabetes mellitus. Ind J Endocrinol Metab. (2018) 22:421–8. doi: 10.4103/ijem.IJEM_585_17, PMID: 30090738 PMC6063173

[12] Fukushima M Enjoji M Kohjima M Sugimoto R Ohta S Kotoh K. Adipose differentiation related protein induces lipid accumulation and lipid droplet formation in hepatic stellate cells. In vitro Cell Dev Biol Anim. (2005) 41:321–4. doi: 10.1007/s11626-005-0002-6, PMID: 16448220

[13] Aouacheri O Saka S Krim M Messaadia A Maidi I. The investigation of the oxidative stress-related parameters in type 2 diabetes mellitus. Can J Diabetes. (2015) 39:44–9. doi: 10.1016/j.jcjd.2014.03.002, PMID: 25065473

[14] Thorens B. GLUT2, glucose sensing and glucose homeostasis. Diabetologia. (2015) 58:221–32. doi: 10.1007/s00125-014-3451-1, PMID: 25421524

[15] Leighton E Sainsbury CA Jones GC. A practical review of C-peptide testing in diabetes. Diabetes Ther. (2017) 8:475–87. doi: 10.1007/s13300-017-0265-4, PMID: 28484968 PMC5446389

[16] Berg JM Tymoczko JL Stryer L. Biochemistry. Section 21.1, Glycogen Breakdown Requires the Interplay of Several Enzymes. 5th ed. New York: W H Freeman (2002).

[17] Samuel VT Shulman GI. The pathogenesis of insulin resistance: integrating signaling pathways and substrate flux. J Clin Invest. (2016) 126:12–22. doi: 10.1172/JCI77812, PMID: 26727229 PMC4701542

[18] Arsenault BJ Lamarche B Després JP. Targeting overconsumption of sugar-sweetened beverages vs. overall poor diet quality for cardiometabolic diseases risk prevention: place your bets! Nutrients. (2017) 9:600–12. doi: 10.3390/nu9060600, PMID: 28608806 PMC5490579

[19] Gardner C Wylie-Rosett J Gidding SS Steffen LM Johnson RK Reader D. Nonnutritive sweeteners: current use and health perspectives: a scientific statement from the American Heart Association and the American Diabetes Association. Diabetes Care. (2012) 35:1798–808. doi: 10.2337/dc12-9002, PMID: 22778165 PMC3402256

[20] Pepino MY Bourne C. Non-nutritive sweeteners, energy balance, and glucose homeostasis. Curr Opin Clin Nutr Metab Care. (2011) 14:391–5. doi: 10.1097/MCO.0b013e3283468e7e, PMID: 21505330 PMC3319034

[21] Suez J Korem T Zeevi D Zilberman-Schapira G Thaiss CA Maza O. Artificial sweeteners induce glucose intolerance by altering the gut microbiota. Nature. (2014) 514:181–6. doi: 10.1038/nature13793 25231862

[22] Cong WN Wang R Cai H Daimon CM Scheibye-Knudsen M Bohr VA. Long-term artificial sweetener acesulfame potassium treatment alters neurometabolic functions in C57BL/6J mice. PLoS One. (2013) 8:e70257. doi: 10.1371/journal.pone.0070257, PMID: 23950916 PMC3737213

[23] De Stefanis D Mastrocola R Nigro D Costelli P Aragno M. Effects of chronic sugar consumption on lipid accumulation and autophagy in the skeletal muscle. Eur J Nutr. (2017) 56:363–73. doi: 10.1007/s00394-015-1086-8, PMID: 26487451

[24] Oguntibeju OO. Type 2 diabetes mellitus, oxidative stress and inflammation: examining the links. Int J Physiol Pathophysiol Pharmacol. (2019) 11:45–63. PMID: 31333808 PMC6628012

[25] Lamb RE Goldstein BJ. Modulating an oxidative-inflammatory cascade: potential new treatment strategy for improving glucose metabolism, insulin resistance, and vascular function. Int J Clin Pract. (2008) 62:1087–95. doi: 10.1111/j.1742-1241.2008.01789.x, PMID: 18489578 PMC2440526

[26] Yung JHM Giacca A. Role of c-Jun N-terminal kinase (JNK) in obesity and type 2 diabetes. Cells. (2020) 9:706. doi: 10.3390/cells9030706, PMID: 32183037 PMC7140703

[27] Margolskee RF Dyer J Kokrashvili Z Salmon KSH Ilegems E Daly K. T1R3 and gustducin in gut sense sugars to regulate expression of Na+−glucose cotransporter 1. Proc Natl Acad Sci USA. (2007) 104:15075–80. doi: 10.1073/pnas.0706678104, PMID: 17724332 PMC1986615

[28] Czech MP. Insulin action and resistance in obesity and type 2 diabetes. Nat Med. (2017) 23:804–14. doi: 10.1038/nm.4350, PMID: 28697184 PMC6048953

[29] Rathinam A Pari L. Myrtenal ameliorates hyperglycemia by enhancing GLUT2 through Akt in the skeletal muscle and liver of diabetic rats. Chem Biol Interact. (2016) 256:161–6. doi: 10.1016/j.cbi.2016.07.009, PMID: 27417257

[30] Leturque A Brot-Laroche E Le Gall M. GLUT2 mutations, translocation, and receptor function in diet sugar managing. Am J Physiol Endocrinol Metab. (2009) 296:E985–92. doi: 10.1152/ajpendo.00004.2009, PMID: 19223655

[31] Pearlman M Obert J Casey L. The association between artificial sweeteners and obesity. Curr Gastroenterol Rep. (2017) 19:64. doi: 10.1007/s11894-017-0602-9 29159583

[32] Knebel B Fahlbusch P Dille M Wahlers N Hartwig S Jacob S. Fatty liver due to increased de novo lipogenesis: alterations in the hepatic peroxisomal proteome. Front Cell Dev Biol. (2019) 7:248. doi: 10.3389/fcell.2019.00248, PMID: 31709254 PMC6823594

[33] McPherson S Hardy T Henderson E Burt AD Day CP Anstee QM. Evidence of NAFLD progression from steatosis to fibrosing-steatohepatitis using paired biopsies: implications for prognosis and clinical management. J Hepatol. (2015) 62:1148–55. doi: 10.1016/j.jhep.2014.11.034, PMID: 25477264

[34] Brenner DA. Molecular pathogenesis of liver fibrosis. Trans Am Clin Climatol Assoc. (2009) 120:361–8. PMID: 19768189 PMC2744540

