# Supplementary figures and images for: High-fat diet mouse model receiving L-glucose supplementations propagates liver injury
Source: Front Nutr. 2024 Dec 13;11:1469952. doi: 10.3389/fnut.2024.1469952 (PMC11687001; doi:10.3389/fnut.2024.1469952)

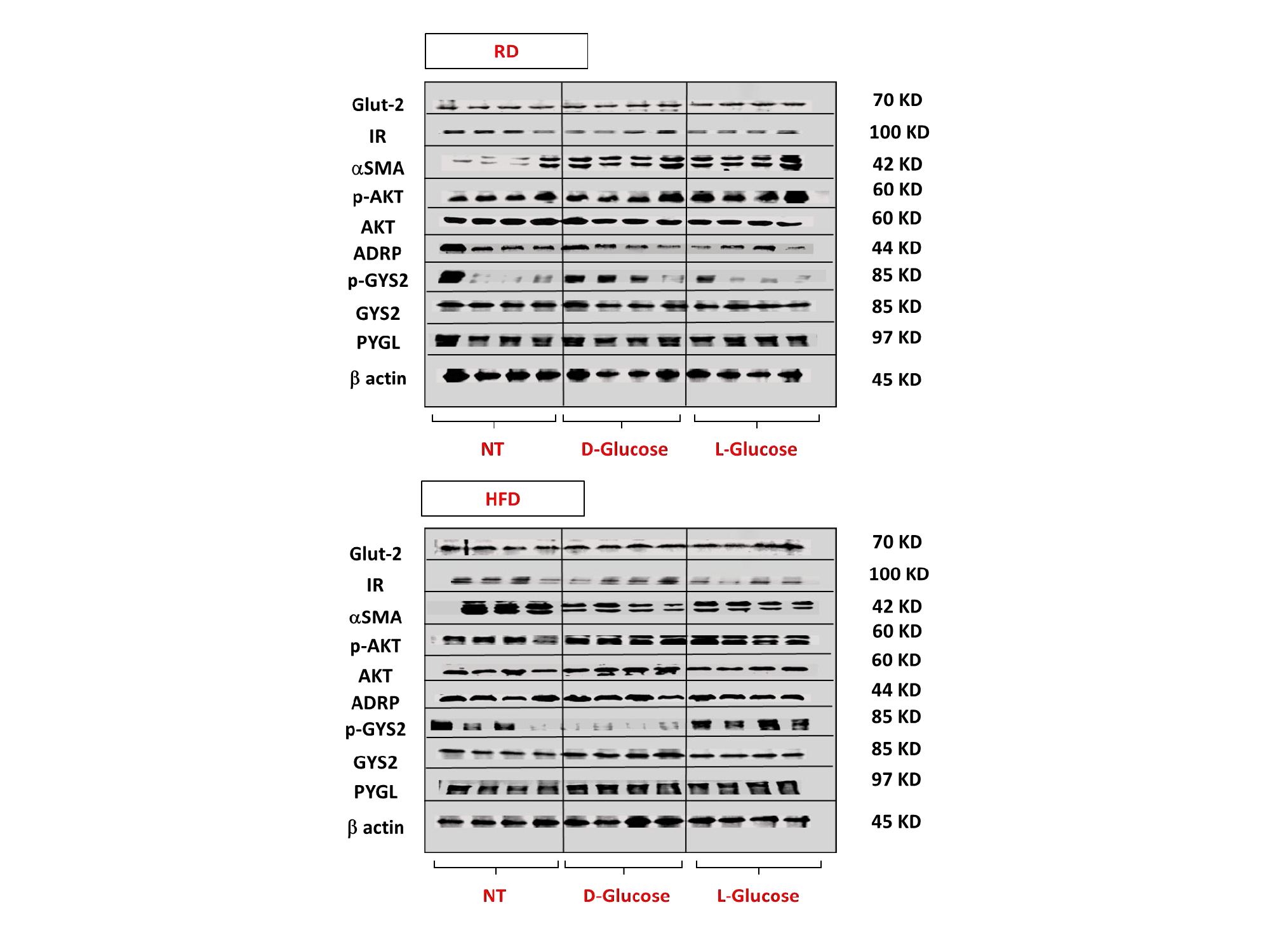

Supplement: Supplementary file 1 [file Image_1.JPEG]

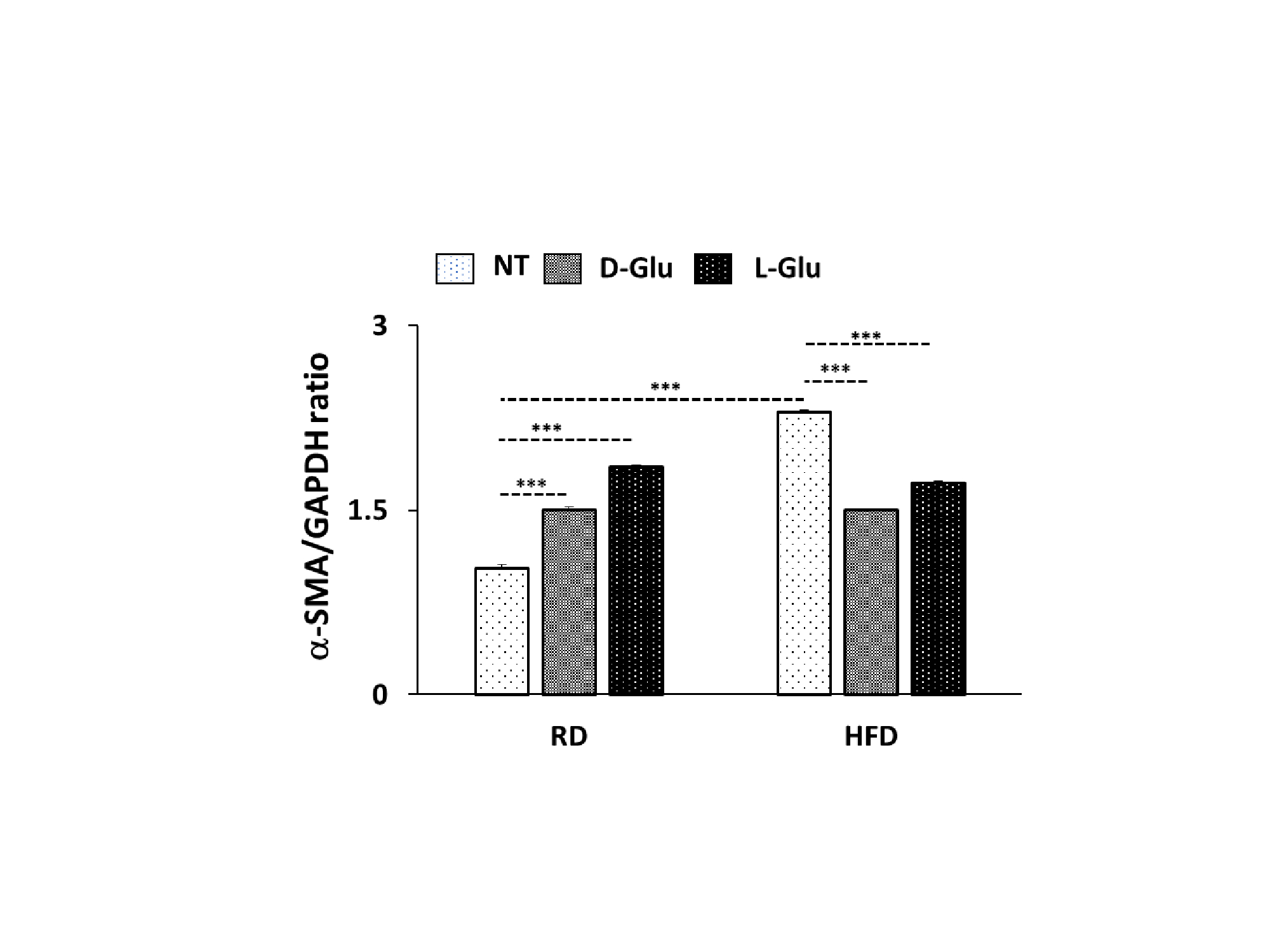

Supplement: Supplementary file 2 [file Image_2.JPEG]
